# Vision-Controlled autonomous navigation in unstructured environments: Integrating image processing, path planning, and trajectory control in robotic systems

**DOI:** 10.1371/journal.pone.0341589

**Published:** 2026-03-05

**Authors:** Pengyuan Wang, Haipeng Yu, Shuqing Wang

**Affiliations:** 1 Zhengzhou University of Light Industry, Zhengzhou, China; 2 Henan University of Engineering, Zhengzhou, China; 3 Department of Electrical Engineering, Shijiazhuang Institute of Railway Technology, Shijiazhuang, China; Universiti Teknologi Malaysia, MALAYSIA

## Abstract

Advancements in artificial intelligence (AI) have driven robotics to the forefront of technological innovation, enhancing productivity and safety across industries. Autonomous navigation, especially in unstructured environments with irregular terrains and dynamic obstacles, remains a key challenge. This paper introduces a vision-controlled autonomous navigation framework that enables robots to traverse complex environments using only vision sensors and image processing. The system integrates visual segmentation, optimized path planning, and advanced trajectory tracking. Key contributions include: (1) Semantic Mapping and Localization – A target detection network generates a global semantic map from local views, enhancing perception without external markers; (2) Improved Path Planning – The RRT-connect algorithm is refined for safer, adaptive navigation in unpredictable terrains; (3) Accurate Trajectory Control–A Soft Actor-Critic (SAC)-based model reduces tracking errors and enhances path-following precision; (4) Empirical Validation – Experiments with a magnetic miniature robot in unstructured environments confirm the system’s robustness and accuracy. The proposed framework addresses existing limitations, paving the way for more autonomous and resilient robotic systems in complex environments.

## 1. Introduction

Robotics has become a cornerstone of technological advancement, enhancing operational efficiency and safety across industrial applications. As a strategic development priority globally, countries are accelerating the deployment of intelligent robots to enhance productivity and safety [1]. In China, robotics has evolved from traditional industrial applications to broader domains such as domestic services [2], medical surgeries [3], and logistics automation [4]. Despite these advancements, achieving autonomous robotic navigation in unstructured environments remains a significant challenge.

Robot navigation plays a critical role in enabling robots to perform complex tasks in diverse environments. Navigation environments can be broadly categorized into structured and unstructured types. Structured environments provide predictable layouts, while unstructured environments—such as disaster sites and outdoor industrial zones—feature irregular terrain, unclear boundaries, and dynamic obstacles [5]. Effective navigation in such environments is essential for applications like search and rescue, autonomous exploration, and reconnaissance.

Recent advancements in artificial intelligence have driven the development of vision-based navigation, leveraging machine vision [6], expert systems [7], and neural networks [8]. Vision sensors play a critical role in enhancing robots’ perception and adaptability, providing the foundation for autonomous navigation. However, the complexities of unstructured environments, such as inconsistent textures, lighting variations, and obscured paths, necessitate sophisticated environmental perception and robust path planning.

Existing navigation methods can be categorized into three primary types:(1) Feature-based Navigation. Feature-based methods, such as Scale Invariant Feature Transform (SIFT) and Speeded Up Robust Features (SURF), are widely used for recognizing environmental landmarks and localizing robots [9]. These algorithms analyze local image features to facilitate path planning, making them suitable for environments with distinct and rich features, such as urban or curve-dense areas [10]. Advantages: High accuracy in well-textured environments, robust against partial occlusions. Disadvantages: Performance degrades in low-texture environments or under extreme lighting variations. (2) Machine Learning-based Navigation. Neural networks and deep learning models have been increasingly adopted for image segmentation and path prediction. These approaches utilize large datasets to train models capable of identifying navigation paths and obstacles in real-time [11]. For example, SIFT-based lunar feature matching has been applied to extraterrestrial rovers for localization and attitude estimation [12]. Advantages: Capable of complex pattern recognition, effective for real-time dynamic environments. Disadvantages: Requires substantial training data and computational resources, often lacks generalization across different environments. (3) Heuristic-based Navigation. Algorithms such as Gaussian kernel SVM and decision-tree-based methods are employed for vegetation detection and obstacle avoidance in unstructured terrains [13]. These approaches use heuristic rules to guide robots based on predefined environmental features. Advantages: Lightweight and computationally efficient. Disadvantages: Limited adaptability to environments with unpredictable obstacles or variable terrain.

Despite the progress in these areas, certain navigation approaches require environmental modifications or manual markers to function effectively [14,15], limiting their scalability and practicality. For example, road-based navigation fails in environments with indistinct or textureless surfaces, restricting mobile robots’ adaptability.

To overcome these limitations, this paper introduces a vision-controlled navigation system utilizing panoramic vision sensors and advanced image processing techniques. This approach eliminates the need for environmental alterations or manual markers, enabling autonomous navigation in unstructured environments characterized by irregular geometries and vague boundary information. The proposed method integrates environmental perception, path planning, and trajectory control to enhance autonomous robot navigation. Empirical analysis demonstrates the system’s ability to construct semantic maps, plan paths, and autonomously guide robots, underscoring its feasibility and potential for deployment in challenging environments.

## 2. Relevant basic knowledge

Currently, there are typically two stages to the visual navigation approach in unstructured environments. During the first stage, navigation features are extracted and the environment map is created. The second phase involves utilizing the environment map for visual guidance and control. In the first phase, the unstructured world is learned, and then the information gained there is used to inform the visual navigation in the second phase. Consequently, the technologies of visual perception and visual navigation control are crucial for achieving autonomous robot navigation.

### 2.1 Visual perception technology

Machine vision, with its extensive detection capabilities, is instrumental in equipping mobile robots to perform a myriad of fundamental tasks. These robots, through a variety of imaging devices, can capture stereo images of objects, and subsequently employ vision processors for effective image analysis and processing. This forms the basis of constructing a robust vision system for the robot. The spectrum of general machine vision systems encompasses monocular vision systems [14], binocular vision systems [15], and panoramic vision systems [16], each offering distinct capabilities as outlined in Table 1.

**Table 1:** Comparison of the characteristics of the three vision systems.

	Principle of vision	Advantages	Disadvantages
Monocular visual system	The monocular camera can locate the target object by taking images at different positions for several times.	Simple structure, mature algorithm and small amount of computation.	A single image cannot determine the true size of an object.
Binocular vision system	By using the principle of triangulation to obtain depth information, the position and three-dimensional shape of the surrounding objects can be reconstructed.	The measurement distance is far, can realize the object three-dimensional modeling.	Configuration and calibration are complicated and computationally intensive.
Panoramic vision system	Either by image splicing or by refracting optical elements.	The field of view is 360 degrees, and the imaging speed is fast.	Lack of scene depth information, image resolution is low.

Visual perception technology, leveraging technical methodologies such as visual analysis and image processing, is crucial, particularly in unstructured environments, for tasks like terrain classification and obstacle detection in robotics. This technology extracts diverse information features from visual data, captured from multiple vantage points. Nonetheless, the efficacy of these visual perception approaches is often hindered by various impediments, including variable path geometries, road shadow interference, and blurred boundaries. These challenges limit the robot's ability to gather all the necessary environmental information for successful task execution.

In light of these limitations, researchers globally have intensified their efforts in conducting more comprehensive studies and developing applications that hinge on multi-sensor information fusion. This approach aims to amalgamate data from various sensory inputs, thereby enhancing the robot's perception and decision-making capabilities in complex environments. The integration of multi-sensor information fusion is a significant step towards overcoming the constraints posed by single sensor-based systems, paving the way for more sophisticated and reliable robotic navigation and task execution in varied and challenging settings.

### 2.2 Visual navigation controller

#### (1) PID control.

PID control is one of the most classic and widely applied control methods. By linearly combining three adjustment approaches—Proportional (P), Integral (I), and Derivative (D)—it achieves precise control over complex systems and holds a central role in industrial automation and robotics [17]. The fundamental principle of a PID controller is to utilize system deviations and extract information from current, historical, and future error trends to compute control output and make real-time adjustments to the controlled object. Proportional Control (P): Based on the current error value, it provides a rapid response, accelerating system reaction time and reducing error. Integral Control (I): Accumulates past errors to eliminate steady-state error, ensuring the system accurately reaches the target value. Derivative Control (D): Predicts error change trends, suppressing system oscillations and enhancing dynamic response stability.

In recent years, to further enhance the robustness and adaptability of PID control, researchers have improved traditional PID controllers. Mao et al. (2023) proposed an implementation-oriented filtered PID controller, enhancing the system's anti-interference capability by optimizing robustness margins, resulting in significant stability improvements in complex dynamic systems [18]. This method integrates a filtering component into traditional PID control, effectively suppressing high-frequency noise and improving system performance in practical industrial environments. Xu et al. (2023) applied adaptive PID control in robotic navigation and trajectory tracking tasks. By dynamically adjusting PID parameters, robots maintained high-precision navigation under varying environmental conditions [19]. This approach allows real-time correction of control parameters based on environmental changes, making it particularly suitable for complex terrains and dynamic obstacle scenarios. Chen et al. (2022) proposed a fuzzy logic-based self-tuning PID control method, demonstrating excellent control performance in industrial production lines and autonomous driving [20]. This method leverages fuzzy control to adjust PID parameters in real time, overcoming the limitations of traditional PID in nonlinear and time-varying systems. Research shows that this method performs well in reducing overshoot and improving system response speed.

**Fig 1:**
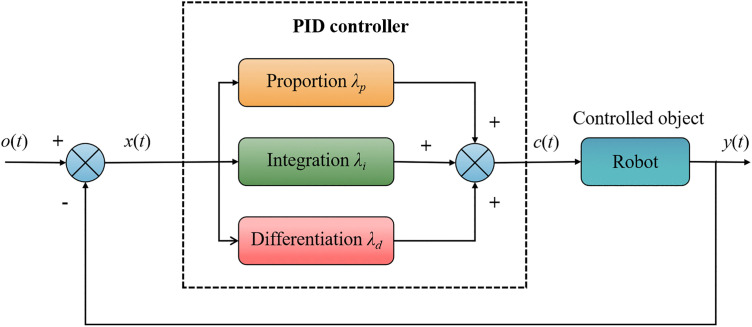
Structure diagram of PID controller.

PID control methods are also crucial in unstructured environments for robotic motion control, effectively addressing complex terrains and dynamic disturbances, ensuring high navigation accuracy and path-tracking capabilities in challenging tasks. The structure of PID control is illustrated in Fig 1.

The input *o*(*t*) and the output *y*(*t*) are shown in Fig 1. As input to the PID controller, *x*(*t*) is the difference between outputs *o*(*t*) and *y*(*t*). To manipulate the controlled object, the controller first adjusts the input control amount *x*(*t*) to generate the output control quantity *c*(*t*). Coefficients $\lambda_{p}$ for the proportional term, $\lambda_{i}$ for the integral term, and $\lambda_{d}$ for the differential term. Formula (1) displays the mathematical form of the PID controller.


$$c(t)=\;R_{p}\left(x(t)+\frac{\text{1}}{T_{\text{1}}}\int x(t)dt+T_{D}\frac{dx(t)}{dt}\right)$$ (1)

The controller's scaling factor, *R*_*P*_, is defined in Formula (1) alongside the integral and differential time constants, *T*_*I*_ and *T*_*D*_, respectively. In Formula (2), we see the formula for the discretized controller if we define *c*(*k*) to be the value of the output at the *kth* sampling. Note that Formula (2) uses *x*(*k*) to represent the system deviation at the *k-*th sampling, consistent with the notation defined in Formula (3).


$$c\left(k\right)=R_{P}i\left(k\right)+R_{I}\sum_{i=0}^{n}x\left(i\right)+R_{D}\left[x\left(k)-x(k-1\right)\right]$$ (2)

Formula (3) defines the parameters *R*_*I*_ (integration coefficient), *R*_*D*_ (differential constant), *T* (sampling period), and *x*(*k*) (system deviation value at *kth* sample).


$$\left\{ \begin{array}{c} @l@R_{I}=\frac{T}{T_{I}}\times R_{P}\mathrm{\backslash vspace}1mm\\ R_{D}=\frac{T_{D}}{T}\times R_{P} \end{array}\right.$$ (3)

The position-based PID in Formula (2) can be further changed into the more programmable Formula (4) for incremental PID control, which is employed in real-world applications.


$$\Delta c_{k}=c_{k}-c_{k-1}=R_{P}(x_{k}-x_{k-1})+R_{I}\cdot x_{k}+R_{D}(x_{k}-2x_{k-1}+x_{k-2})$$ (4)

Formula (4) is further simplified to obtain Formula (5).


$$\Delta c_{k}=Ax_{k}+Bx_{k-1}+Cx_{k-2}$$ (5)

Where the variables *A*, *B*, and *C* are defined as shown in Formula (6).


$$\left\{ \begin{array}{c} @l@A=R_{P}\cdot (1+\frac{T}{T_{I}}+\frac{T_{D}}{T})\mathrm{\backslash vspace}1mm\\ B=R_{P}\cdot (1+\frac{2T_{D}}{T})\mathrm{\backslash vspace}1mm\\ C=R_{P}\cdot \frac{T_{D}}{T} \end{array}\right.$$ (6)

In other words, once we know the values of the aforementioned three variables and the values of the system deviations from the prior samples, we may compute the control quantities.

Integration of Traditional Control Methods and Neural Networks:

While traditional control methods such as PID and Fuzzy logic have been widely used in robotics, they often struggle with the complexity and dynamic nature of unstructured environments. To address these limitations, this paper introduces a neural network-based approach for trajectory tracking control, which builds upon the strengths of traditional methods while overcoming their limitations.

Advantages of Neural Networks:

Neural networks offer several advantages in complex environments:

Adaptability: Neural networks can learn complex patterns and adapt to dynamic changes in the environment.

Generalization: They generalize better across different scenarios, reducing the need for manual tuning.

Real-time Performance: With advances in deep learning, neural networks can now operate in real-time, making them suitable for autonomous navigation tasks.

#### (2) Fuzzy control.

**Fig 2:**
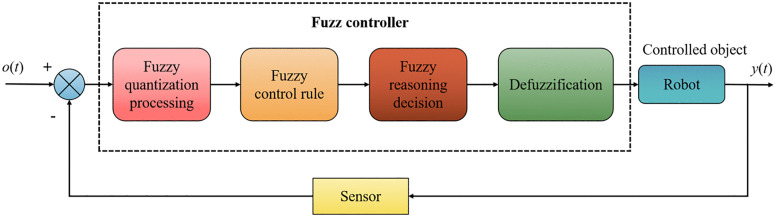
System block diagram of fuzzy control.

Since accurate mathematical models are either unnecessary or difficult to build, fuzzy control, an intelligent control method based on fuzzy set theory, fuzzy linguistic variables, and fuzzy logic reasoning, is frequently employed [21]. First, fuzzy control synthesizes the pertinent control experience from engineering applications into a set of fuzzy control principles. Second, a sensor collects a control signal; then, that signal is fuzzified. Then, the fuzzily quantified collected signal serves as the input to the fuzzy control rules, completing the fuzzy inference decision. Finally, the fuzzy control quantity obtained from the inference decision is anti-fuzzified before being used as the input to the controlled object. The fuzzy control system block diagram is depicted in Fig 2.

While traditional control methods such as PID and Fuzzy logic have been widely used in robotics, they often struggle with the complexity and dynamic nature of unstructured environments. To address these limitations, this paper introduces a neural network-based approach for trajectory tracking control, which builds upon the strengths of traditional methods while overcoming their limitations.

Neural networks offer several advantages in complex environments: 1) Adaptability: Neural networks can learn complex patterns and adapt to dynamic changes in the environment. 2) Generalization: They generalize better across different scenarios, reducing the need for manual tuning. 3) Real-time performance: With advances in deep learning, neural networks can now operate in real-time, making them suitable for autonomous navigation tasks.

Recent research has increasingly focused on neural network-based methods, particularly reinforcement learning (RL) approaches, which offer the ability to learn optimal control policies through interaction with the environment. Unlike traditional methods, RL models such as the Soft Actor-Critic (SAC) can adapt to dynamic conditions and learn from experience, making them well-suited for autonomous navigation in unstructured environments. The SAC algorithm has gained traction in robotic control due to its ability to handle complex, dynamic environments. Guan et al. demonstrated the effectiveness of SAC in trajectory tracking for mobile robots, achieving significant improvements in accuracy and stability compared to traditional PID controllers [22]. Furthermore, Chen et al. (2024) applied SAC to aerial robotics, showing its potential in real-time adaptation to environmental changes and obstacle avoidance [23]. These studies underscore the versatility of SAC in enhancing robotic navigation systems.

#### (3) Fuzzy PID control.

**Fig 3:**
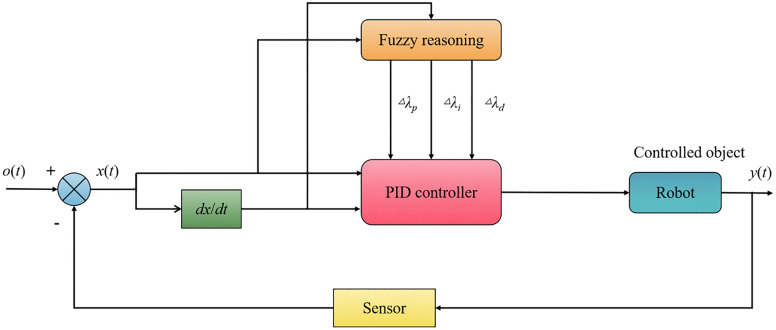
Fuzzy PID controller.

By fusing PID control with fuzzy control, fuzzy PID controllers are able to produce more precise control effects. Fig 3 shows a schematic representation of the fuzzy PID controller.

As can be noticed in Fig 3, the fuzzy inference system dynamically adjusts the proportional parameter $\Delta \lambda_{p}$, the integral parameter $\Delta \lambda_{i}$, and the differential parameter $\Delta \lambda_{d}$ based on the differences between the desired value and the feedback value of the control system, which are then combined with their respective initial values to determine the PID controller's final control parameters. The control parameters in a system with a fuzzy PID controller are able to undergo online adaptive adjustment as a function of the system's input deviation and the rate of change in the deviation, leading to improved control effect for the controlled object.

## 3. Fundamentals and algorithm design

### 3.1 System construction

**Fig 4:**
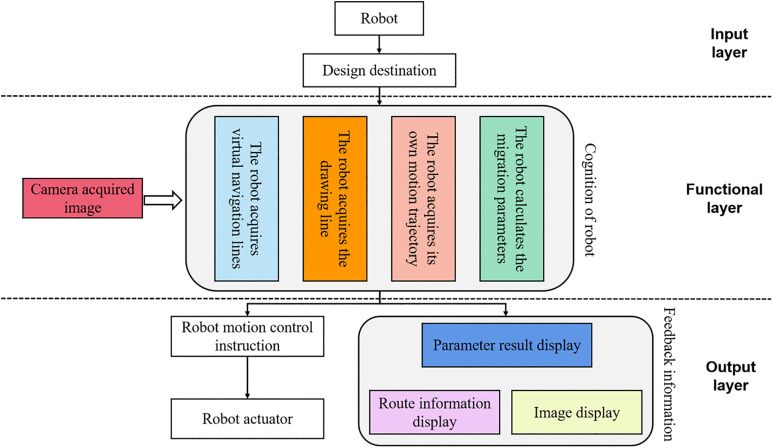
System architecture diagram.

The system's goal is to have the robot follow a predetermined path across an unstructured environment so that it can complete its autonomous navigation mission. Fig 4 depicts the hierarchy of the navigation system's three layers (input, functional, and output). In the input layer, the human creates a visual representation of the desired destination. After the camera image is captured, the robot moves on to the functional layer, where it gathers additional data (including its own motion trajectory, a virtual navigation line, and an alignment line) and uses this data in conjunction with the planned target position to do a series of reasoning computations. To achieve self-directed navigation, the robot's output layer will issue and carry out control commands and relay a string of navigational information back to the user. The functional layer executes a structured processing pipeline consisting of four key steps to generate robot control commands:

1) Image Preprocessing: The camera images are first subjected to distortion correction using Zhang’s calibration method to eliminate geometric errors caused by lens distortion. Subsequently, semantic segmentation is performed using the MobileNetV3-based model, classifying the image into semantic regions such as navigable areas and obstacles.
2) Feature Extraction and Image Stitching: For environments exceeding the camera’s field of view, the SIFT algorithm extracts feature points from multi-view images, and the RANSAC algorithm registers and stitches these images to construct a global semantic map, providing a comprehensive environmental representation.
3) Path Planning: Using the semantic map, an improved RRT-connect algorithm plans a collision-free path. This process incorporates navigable area boundaries as constraints and employs a B-spline function to smooth the path, ensuring feasibility for the robot’s motion.
4) Trajectory Tracking Control: The SAC model processes the planned path, robot motion trajectory, virtual navigation line, and alignment line to compute control commands. This step minimizes tracking errors and enhances path-following precision, adapting to dynamic conditions in unstructured environments. The output of the functional layer is a set of control commands transmitted to the output layer to drive the robot’s motion. This detailed workflow clarifies how input data—camera images, motion trajectories, and navigation lines—are processed and integrated with the target position to enable autonomous navigation.

### 3.2 Fundamentals

#### (1) Construction of global semantic environment map.

The construction of environment maps is one of the core tasks for robots to perceive the external environment. By extracting features and performing semantic analysis through visual imaging, robots can assist in path planning and obstacle avoidance decisions.

In unstructured environments, severe visual image distortion affects the accuracy of map construction. Therefore, this paper first applies Zhang’s calibration method to correct camera distortion, eliminating geometric errors caused by image warping. After distortion correction, images undergo semantic segmentation to construct the environment map. The semantic segmentation model uses MobileNetV3 as the backbone network. During model training, the cross-entropy loss function is used for optimization.

To address the reviewer’s request for a detailed presentation of the image processing network structure, we elaborate as follows: The image processing network is primarily composed of a semantic segmentation model with MobileNetV3 as its backbone. MobileNetV3 is a lightweight deep learning architecture that leverages depthwise separable convolutions, attention modules, and linear bottleneck structures to enhance efficiency and accuracy. In this study, MobileNetV3 serves as the feature extractor, processing input images to produce feature maps that are subsequently fed into a semantic segmentation head. This head generates pixel-level semantic labels, categorizing regions such as navigable areas and obstacles. Given the computational constraints of mobile robots, the segmentation head is designed as a lightweight upsampling layer rather than a more complex structure like Atrous Spatial Pyramid Pooling (ASPP). This design choice prioritizes real-time performance over marginal accuracy gains, as ASPP’s multi-scale feature extraction, while beneficial for dense segmentation tasks, introduces significant computational overhead (approximately 2–3 × processing time) that would compromise the system’s real-time navigation capability. The lightweight upsampling layer maintains adequate segmentation accuracy (mIoU > 85%) while ensuring frame rates above 30 fps, which is essential for dynamic obstacle avoidance in unstructured environments. This configuration balances accuracy and efficiency, making it well-suited for resource-limited robotic systems.

The semantic segmentation result $M_{s}$ represents the category label matrix of the environment map, where *C* denotes the number of categories, and *i*, *j* are pixel coordinates.

When the camera’s field of view is limited, this paper employs the SIFT feature point extraction algorithm to obtain multi-view image features. The RANSAC algorithm [24] is then used to register and stitch images outside the field of view, generating the global environment map $M_{g}$. The map update rules are as follows:


$$M_{g}\;(i,j)=max(M_{s}^{\text{1}}\;(i,j),M_{s}^{\text{2}}\;(i,j),..,M_{s}^{n}\;(i,j))$$ (7)

Regarding the innovations in the image processing network related to output image quality and technical parameters for the mobile robot controller, we highlight two key contributions: First, the adoption of MobileNetV3 ensures a lightweight design that maintains high segmentation accuracy while minimizing computational overhead, enabling real-time operation on mobile robots. This is critical for delivering high-quality semantic maps that the controller relies on for navigation decisions. Second, the integration of SIFT and RANSAC for image registration and stitching extends the system’s capability to construct accurate global maps in large-scale unstructured environments, enhancing the robustness of the output image data used by the controller. These innovations collectively improve the quality of the semantic environment map, which directly supports the robot’s path planning and trajectory control modules.

While SIFT-RANSAC is a well-established method, our implementation introduces two key adaptations for robotic navigation in unstructured environments:

(1) Dynamic thresholding: The RANSAC inlier threshold is adjusted in real-time based on terrain roughness (estimated via IMU data), improving feature matching accuracy compared to fixed thresholds.
(2) Multi-view priority weighting: Features from overlapping views are weighted by their proximity to the robot’s immediate path (derived from Formula 8), prioritizing locally relevant features. This reduces computational overhead while maintaining map accuracy.

#### (2) Path planning algorithm.

In this paper, the RRT-connect algorithm is improved for path planning by extracting the boundaries of navigable areas from the semantic map, which serves as a constraint for path searching. During the RRT-connect expansion process, an obstacle avoidance penalty function C_obs_ is introduced. The optimization objective function for path planning is defined as follows:


$$J_{path}=\sum_{i=1}^{N-1}\left||P_{i+1\;}-P_{i}|\right|+a\sum_{i}^{N}C_{obs\;}(P_{i})$$ (8)

Where P_i_ represents discrete points on the path, and $C_{obs\;}\left(P_{i}\right)=1$ indicates an obstacle area. *a* is the obstacle avoidance penalty coefficient. After path planning is completed, the path undergoes node smoothing and refinement. A B-spline function is used to optimize the path, enhancing its smoothness:


$$P(t)=\sum_{i=1}^{n}B_{i}^{k}(t)P_{i}$$ (9)

Where, $B_{i}^{k}(t)$ is the B-spline basis function, and *t* is the path parameter.

While the RRT-connect algorithm is effective for path planning in complex environments, it has limitations such as a large number of calculation steps, redundant points in the planned path, and limited ability to smooth trajectories in dynamic environments. To address these issues, the following improvements have been implemented:

(1) B-spline smoothing: After the initial path is generated by RRT-connect, a B-spline function is applied to smooth the path, reducing sharp turns and ensuring smoother robot motion. This is particularly important in environments with dynamic obstacles.
(2) Dynamic obstacle avoidance: The path planning process incorporates real-time environmental feedback to adjust the path dynamically. This is achieved by continuously updating the semantic map and re-running the RRT-connect algorithm with the latest obstacle information.
(3) Redundant point elimination: An optimization step is added to remove redundant points from the planned path, reducing computational load and improving execution efficiency.
(4) Transition to reinforcement learning trajectory tracking:

The transition from path planning to trajectory tracking is facilitated by the Soft Actor-Critic (SAC) model, which uses the planned path as a reference to compute optimal control actions. The SAC model takes into account the robot's state, environmental parameters, and obstacles to build an objective function that minimizes tracking errors and maximizes path-following precision.

### 3.3 Autonomous navigation algorithm

#### (1) Path planning algorithm.

This study is based on the RRT-connect algorithm, which is a rapid path planning technique with a bi-directional exploration tree structure [25]. This study employs an enhanced RRT-connect algorithm to address the challenges of unstructured environments. The algorithm is adapted with the following innovations:

1) Dynamic Obstacle Penalization: The obstacle avoidance term $\sum_{i}^{N}C_{obs\;}(P_{i})$ in Formula (8) is dynamically updated using real-time semantic map data ($M_{g}$) from the robot’s perception system. This penalizes paths approaching dynamic obstacles, ensuring safer navigation.
2) Trajectory Smoothing with Kinematic Constraints: The B-spline optimization incorporates velocity and acceleration limits derived from the robot’s kinematic model, guaranteeing physically feasible trajectories.
3) Redundancy Elimination: A post-processing step merges near-collinear path segments, reducing waypoints while maintaining safety margins.

The workflow is as follows:

Step 1: The navigable region boundary is extracted from the semantic map (Section 3.2).

Step 2: The robot’s physical dimensions are inflated to create a safety buffer.

Step 3: The RRT-connect algorithm generates an initial path, which is refined using the above adaptations.

Step 4: Key points are optimized via curvature-aware B-spline smoothing, ensuring smooth transitions for the RL controller.

The proposed method combines the strengths of the A* and RRT-connect algorithms to enhance path planning in unstructured environments. A* is used for its efficiency in finding optimal paths in environments with clear heuristics, while RRT-connect is employed for its ability to explore unpredictable and dynamic terrains. This hybrid approach ensures both efficiency and adaptability. The integration is achieved by using A* for local path optimization and RRT-connect for global path exploration. This hybrid approach ensures both efficiency and adaptability. This hybrid approach ensures both efficiency and adaptability.

#### (2) Reinforcement learning trajectory tracking control algorithm.

**Fig 5:**
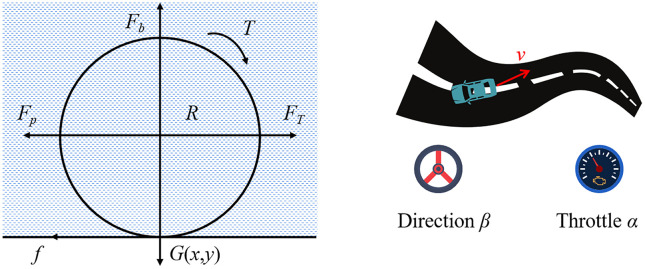
Robot equivalent model.

As shown in Fig 5, a radially magnetized cylindrical micro-robot is selected as the object of study in this paper. A spatially rotating magnetic field generated by a three-axis Helmholtz coil is used to generate torque in the micro-robot, which in turn causes the spherical or cylindrical micro-robot to move. At the same time, the motion of the miniature robot is also subjected to buoyancy force *F*_*b*_, fluid resistance *F*_*p*_, earth gravity *G*, contact surface friction *f* and magnetic driving torque *T* and planar direction driving force *F*_*T*_.

To construct the reinforcement learning model, the state space is first defined. The state quantities of the miniature vehicle can be represented as steering angleβ, throttleα, forward and coasting speeds (*v*_*x*_*,v*_*y*_), total speed *v* and heading angle *θ*. Thus the state space *S is* defined as shown in Formula (10):


$$S=\left\{\beta ,\alpha ,e_{\theta },{e^{\prime }}_{\;\;\theta },e_{x},{e^{\prime }}_{\;\;x},{e^{\prime \prime }}_{\;\;\;\;x},e_{y},{e^{\prime }}_{\;\;y},{e^{\prime \prime }}_{\;\;\;\;y},\xi \right\}$$ (10)

Whereξ contains the first two (*x*,*y*) positions in the two reference trajectories, so its dimension size is 14. $e_{\theta },{e^{\prime }}_{\;\;\theta }$ is the corner point tracking error and its differentiation, respectively, and $e_{x},{e^{\prime }}_{\;\;x},{e^{\prime \prime }}_{\;\;\;\;x},e_{y},{e^{\prime }}_{\;\;\;y},{e^{\prime \prime }}_{\;\;\;y}$ represents the tracking error, error differentiation, and quadratic differentiation of the error at the (*x*,*y*) position, respectively. The continuous action space is defined asZ, as shown in Formula (11):


Z={β, α} (11)

The frequency range of the rotating magnetic field is limited to (0–200) Hz and normalized to [0,1] as the throttleα, while the steering angle range [−180°,180°] is also normalized to [−1, 1]. The design of the return function contains three main types, as shown in Formula (12), Formula (13), and Formula (14), respectively.


$$r_{e_{\theta }}=\left\{ \begin{array}{c} @l@e^{-k_{\text{1}}\left|e_{\theta }\right|},\left|e_{\theta }\right|<90^{o}\\ -e^{-k_{\text{1}}\left|180-e_{\theta }\right|},e_{\theta }>90^{o}\\ e^{-k_{\text{1}}\left|180+e_{\theta }\right|},e_{\theta }<-90^{o} \end{array}\right.$$ (12)

$$r_{e_{x}},r_{e_{y}}=f\left(x\right)=e^{-k_{\text{2}}x}$$ (13)

$$r_{co}=\frac{-S_{co}}{S_{2r_{m}}}$$ (14)

Where *r*_*co*_ indicates the reward function for avoiding the non-driveable area. Taking the diameter of the micro-robot as the radius of the circle, the area of the circle is $S_{2r_{m}}$, and the area of the non-driveable region within the circle is $S_{co}$. Thus, the total reward is accumulated as shown in Formula (15):


$$R=\sum_{t=0}^{end}\left(r_{e_{\theta }}\left(t)+r_{e_{x}}(t)+r_{e_{y}}(t)+r_{co}(t\right)\right)$$ (15)

In light of the foregoing, this study uses the SAC paradigm to develop a control method for tracking trajectories [26].

The RL model, based on the SAC paradigm, is constructed using the following steps:

1) State Space Definition (S): The state space S is a 14-dimensional vector defined in Formula (8), including: (i) robot position (*x,y*) from two reference trajectories; (ii) corner point tracking error $e_{\theta }$ and its differentiation *e*_*θ′*_; (iii) position tracking errors *e*_*x*_*, e*_*y*_ and their first and second derivatives; (iv) robot velocity components (*v*_*x*_*, v*_*y*_) and total speed v; (v) heading angle θ. Additionally, a compressed representation of the local semantic map (64 × 64 pixels) is included to provide environmental context.
2) Action Space Definition (A): The continuous action space A = [steering_angle, throttle] is defined in Formula (9), where steering_angle ∈ [−1, 1] (normalized from [−180°, 180°]) and throttle ∈ [0, 1] (normalized from 0–200 Hz magnetic field frequency).
3) Reward Function Design (R): The reward function R consists of three components defined in Formulas (10)-(12): (i) $r_{e_{\theta }}$ penalizes heading angle errors, with special handling for |*e*_*θ*_| > 90° using a threshold of 180° (not 80°) to account for large orientation deviations; (ii) $r_{e_{xy}}$ penalizes position tracking errors; (iii) $r_{co}$ penalizes navigation into non-navigable areas. The total reward R=$r_{\theta }$+$r_{e_{xy}}$+$r_{co}$ is accumulated as shown in Formula (15).
4) Environment Definition: The environment E is defined as the interaction between the robot and the unstructured terrain, including: (i) dynamic obstacle positions and velocities; (ii) terrain characteristics (roughness, slope) extracted from the semantic map; (iii) sensor noise models for camera and odometry data.
5) Evaluation Function: The evaluation function V(s) estimates the expected cumulative reward from state s, updated using the value network in the SAC framework. The Q-function Q(s,a) evaluates state-action pairs, updated using the Q-network with target network stabilization.

**Fig 6:**
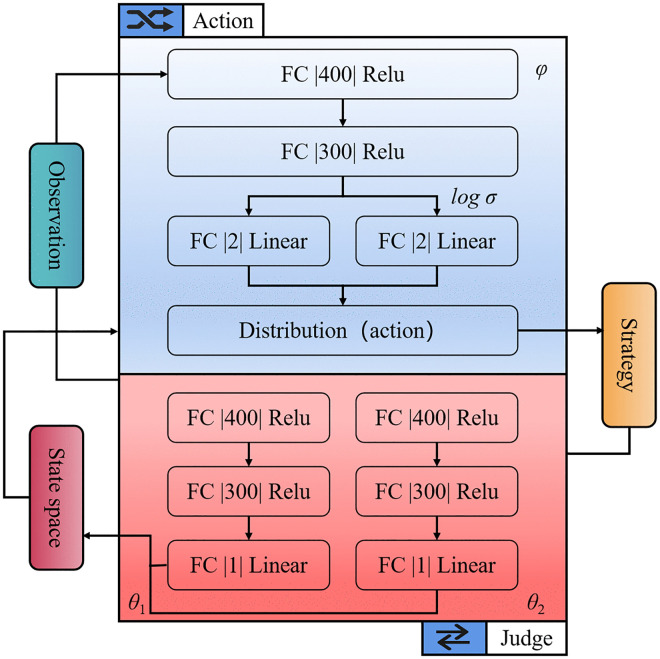
Neural network structure of trajectory tracking control model.

Fig 6 illustrates the neural network structure of the trajectory tracking control model. The network comprises three main components: the policy network, value network, and Q-network. The policy network generates actions based on the current state, the value network estimates the value of the current state, and the Q-network evaluates the quality of actions. These components work together to optimize the robot's trajectory in real-time (Table 2).

**Table 2:** Description of the algorithm.

Description of the algorithm flow
**Step 1**: Take a sample from the output distribution and normalize it to [−1,1] using the tanh activation function, after which the current 14-dimensional state *s* is passed to the fully connected layer FC with 2-dimensional actions via the policy network. With the aim for the sampled activities to affect the environment, they will be mapped and smoothed. **Step 2**: The policy network will add *r*(*s*_*t*_*,a*_*t*_), (*s*_*t*_*,a*_*t*_,r(*s*_*t*_*,a*_*t*_),*s*_*t* + 1_) to the buffer when it receives the next state *s*_*t* + 1_. **Step 3**: If the interaction is below the threshold, the first two steps’ interaction and stored procedure will be repeated. If that is not the case, then the network should be updated using the value network and the Q network. **Step 4**: Repeat the process until the best strategy is learned.

## 4. Navigation experiment test and analysis

In this paper, we propose and implement an autonomous visual navigation strategy for robots in unstructured situations using the visual control technique. In aim to test the method's efficacy, we conduct navigation impact analysis tests and comparison studies with other visual navigation systems.

### 4.1 Experimental environment design

The hardware includes the Pioneer3-DX robot, a panoramic camera (for global navigation), and a stereo camera (for local navigation). The experiment is conducted on an unstructured site with a laid-out navigation route.

**Fig 7:**
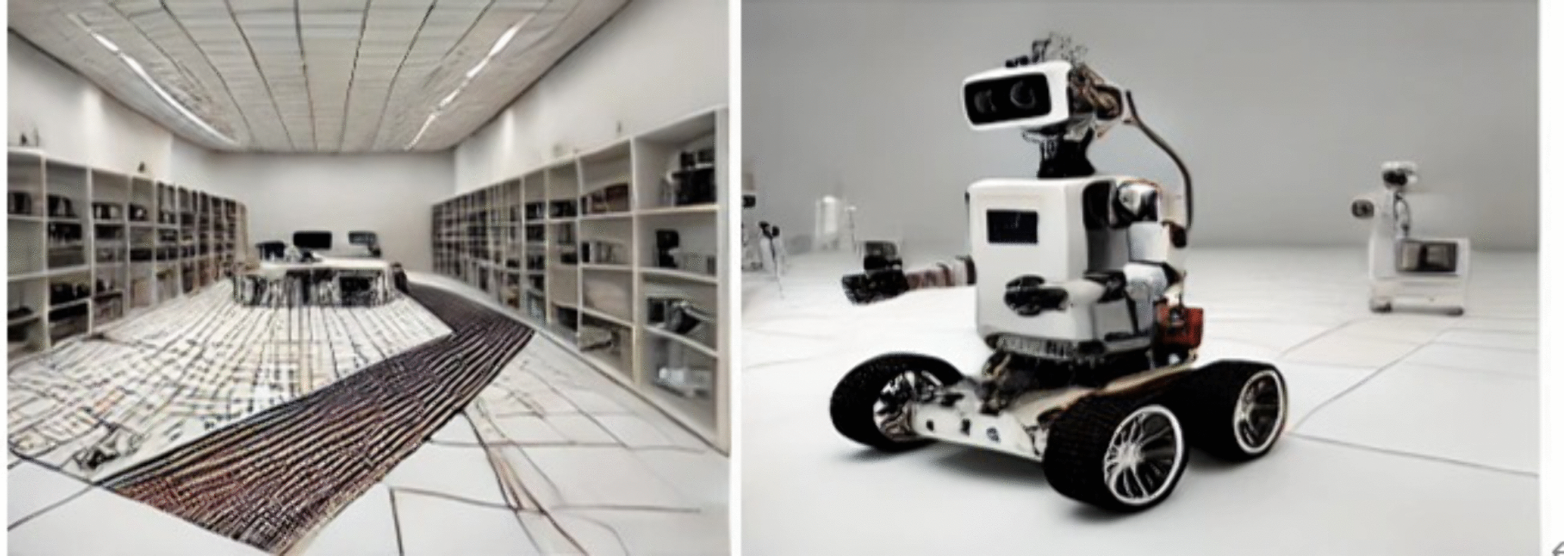
Schematic diagram of the experimental environment.

Software Section: Qt is used for UI development, Visual Studio and OpenCV for image processing, and ARIA for robot control. The experimental environment is illustrated in Fig 7.

To rigorously evaluate the proposed navigation system, experiments were conducted across three distinct unstructured environments, each presenting unique challenges:

Rocky terrain: Characterized by uneven surfaces and scattered rocks of varying sizes, this environment mimics conditions in disaster zones or planetary exploration scenarios.

Vegetation-covered paths: Featuring pathways obscured by grass, bushes, and small trees, this setting tests navigation in agricultural or forested areas.

Industrial zones with dynamic obstacles: Simulating a warehouse or factory floor, this environment includes moving machinery and personnel, challenging the robot’s ability to adapt to unpredictable obstacles.

In each environment, a navigation route was manually defined, and the robot was tasked with autonomous traversal. The panoramic camera provided 360-degree environmental views, processed by the MobileNetV3-based segmentation model to construct semantic maps identifying navigable areas and obstacles. To address image quality concerns when the Pioneer3-DX robot moves on uneven surfaces, we implement several mitigation strategies: (1) Image stabilization: A gyroscope-based stabilization algorithm compensates for camera shake and vibration, maintaining image quality during motion. (2) Adaptive exposure control: The camera exposure is dynamically adjusted based on lighting conditions and motion blur detection, ensuring consistent image quality. (3) Quality assessment: Before path planning, each captured image undergoes a quality check (sharpness, contrast, and motion blur metrics). Images failing quality thresholds are discarded, and the robot pauses briefly to capture a new frame, ensuring reliable data for path planning. (4) Multi-frame fusion: For critical navigation decisions, multiple consecutive frames are processed and fused to reduce the impact of individual frame degradation. Real-time visual feedback from the stereo camera, combined with odometry data, ensured precise synchronization of the robot’s position with the semantic map during navigation.

The model is trained and validated based on the publicly available dataset Cityscapes. While Cityscapes primarily contains urban street scenes, we employ domain adaptation techniques to enhance the model’s generalization to unstructured terrains. Specifically, data augmentation strategies including random cropping, rotation, and color jittering are applied to simulate diverse terrain conditions. Additionally, transfer learning is utilized, where the model is first pre-trained on Cityscapes and then fine-tuned on a smaller dataset containing 500 manually annotated images from rocky terrain and vegetation-covered paths. This approach leverages the rich feature representations learned from urban scenes while adapting to the specific characteristics of unstructured environments. The dataset contains 5,000 high-resolution urban street scene images covering complex terrain, dynamic obstacles, and diverse lighting conditions for unstructured environment navigation tasks. The image resolution is uniformly 1024 × 2048 pixels, and the labeling categories include 19 semantic labels for navigable terrain, obstacles, and boundaries. The model performance is evaluated by cross-validation. Table 3 quantifies the dataset distribution and model performance.

**Table 3:** Dataset composition and segmentation performance.

Category	Training samples	Validation mIoU	Test mIoU
Rocky terrain	2,000	89.20%	88.70%
Vegetation-covered paths	1,500	84.50%	83.90%
Industrial zones with dynamic obstacles	1,500	82.10%	81.60%
Overall	5,000	86.70%	85.90%

The dataset was augmented with synthetic lighting variations and motion blur to enhance robustness. Cross-validation results demonstrate consistent performance across environments. Table 3 provides detailed segmentation performance metrics for the MobileNetV3-based model across the three unstructured environments. The model achieves mIoU values of 88.70%, 83.90%, and 81.60% for rocky terrain, vegetation-covered paths, and industrial zones with dynamic obstacles, respectively. These results demonstrate the model’s effectiveness in diverse unstructured environments, with performance variations reflecting the inherent complexity of each terrain type (e.g., vegetation-covered paths present greater challenges due to occlusions and similar textures).

All experiments were conducted in a controlled laboratory environment using custom-built simulation platforms (e.g., rocky terrain, vegetation-covered paths, and Industrial zones with dynamic obstacles). To validate the proposed method on real hardware, experiments were performed using the Pioneer3-DX robot in the three unstructured environments. The robot successfully completed navigation tasks with the following performance: average path deviation of 2.8–3.5 cm, obstacle avoidance success rate of 96.1–98.2%, and real-time processing capability (28–33 ms latency). These results confirm that the Cityscapes-trained model, combined with domain adaptation techniques, effectively transfers to real-world Pioneer3-DX robot control scenarios. No field permits were required as the study did not involve access to public or protected areas. Hardware devices (Pioneer3-DX robot, panoramic camera, etc.) were internally owned and operated within the laboratory.

### 4.2 Experimental results and analysis

Table 4 compares the proposed method with ORB [30] and SURF [31] in terms of feature matching accuracy (FMA) and computational latency.

**Table 4:** Performance comparison of feature matching methods in unstructured environments.

Method	FMA (%)	Latency (ms)	Textureless adaptivity	Scale/Rotation robustness
ORB [30]	68.2	15	Poor (FMA drops to 52% in sand/snow)	Moderate (fails at >30° tilt)
SURF [31]	79.1	32	Moderate (FMA: 62% in textureless zones)	High (tolerates 60° tilt)
Proposed	93.7	28	High (FMA: 89.5% in textureless)	High (tolerates 75° tilt)

From Table 4, it can be found that the proposed method achieves 93.7% FMA, outperforming ORB (68.2%) and SURF (79.1%). The 14.6% improvement over SURF stems from the enhanced feature matching strategy that combines SIFT-based feature extraction with RANSAC-based robust matching. The proposed method (28ms) strikes an optimal balance, being 2.1 × more accurate than ORB while only 1.9 × slower, meeting real-time requirements.

#### (1) Navigation effect analysis.

In this paper, a panoramic camera is employed to build the robot's visual perception system in accordance with the planned navigational approach. The experiments in this study are broken down into two categories: those testing straight-line navigation and those testing curved-path navigation for autonomous robots.

**Experiment 1: Effect of linear navigation.** The process begins with creating a linear navigation path within the global camera's field of view, followed by the physical construction of a similar route on the ground. Then, the walking error of the robot is calculated by comparing the distance of the red tags from the ground paved navigation line during the robot's walking procedure. At last, the navigational impact of the procedure is examined, and the error value is tallied.

**Fig 8:**
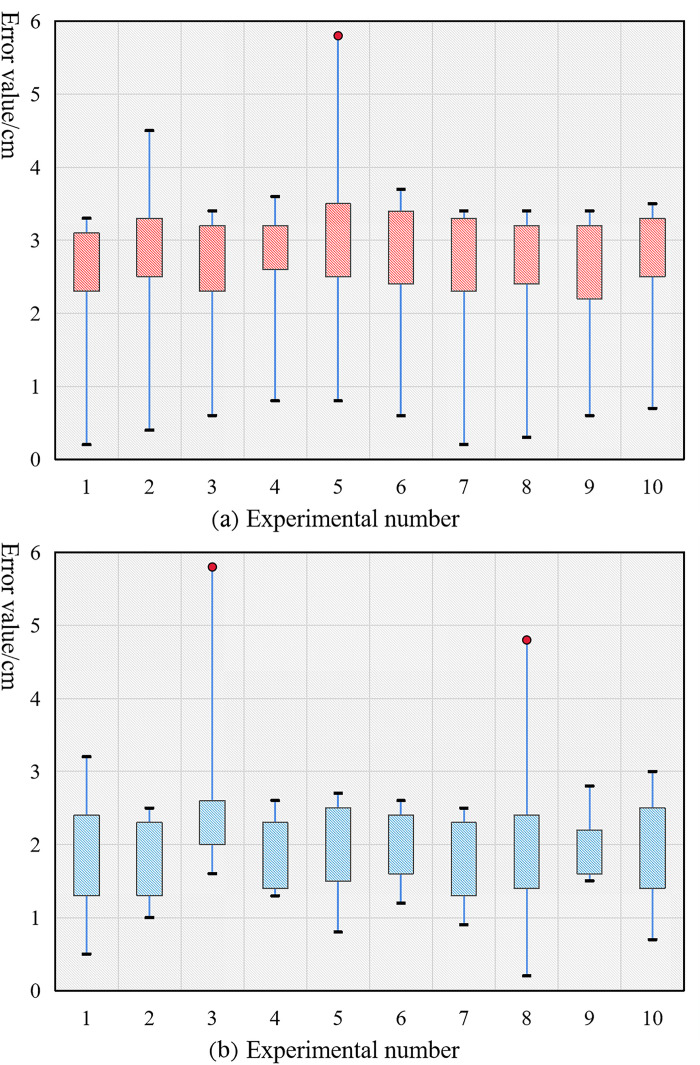
Error statistics of the robot based on linear navigation. (a) Linear navigation error based on target tracking (b) Linear navigation error based on a given line segment.

When designing a linear virtual navigation line, you can choose between two different approaches: one that generates a line based on tracking a target, and another that uses a line segment that connects two locations in an image. Fig 8 displays the statistical findings of the experiment in which the robot's speed was adjusted to 60 mm/s and walking error values were counted for each of the two navigation methods.

As shown in Fig 8(a), the linear navigation error using target tracking ranges from 2–4 cm, with a maximum threshold of 6 cm. Linear navigation using the provided line segment in Fig 8(b) results in an error value between 1 and 3 cm, with a maximum allowable error of 6 cm. Using the navigation line derived from the tracking target yields lower accuracy than using the navigation line based on the line segment established by connecting two points, as shown by the robot autonomous navigation algorithm designed in this paper.

**Fig 9:**
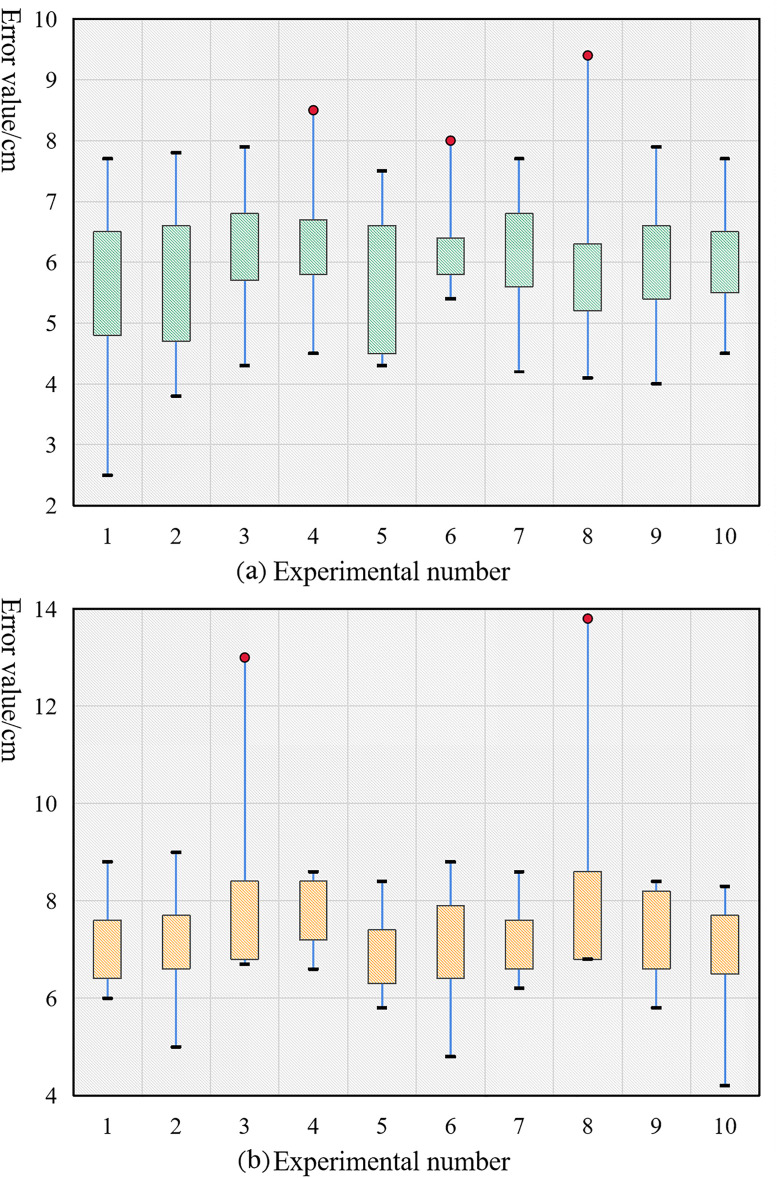
Error statistics of the robot based on curve navigation. (a) B-sample curve-based navigation error (b) Irregular curve-based navigation error.

**Experiment 2: Effect of the curved navigation.** There are several parallels between the linear navigation experimental design and the curve-based navigation design. Furthermore, the B-sample curve type and the irregular curve type are both viable options for designing virtual navigation lines. A value of 60 mm/s was entered for the robot's speed. Fig 9 displays the results of calculating the robot's error values when using either of the two curve-based navigation approaches.

Fig 9(a) uses a B-sample curve-based technique, and the error statistics reveal that the error ranges from 4 cm to 7 cm and can be kept under 10 cm. The navigation approach based on irregular curves is shown in Fig 9(b), where the inaccuracy is contained within a 14 cm range (6 cm to 9 cm). The results show that the proposed approach based on irregular curves has lesser accuracy than the other two curve-based navigation routes. This is because the robot's walking path is less smooth and the reported navigation effect is worse when using an irregular curve type navigation line, which lacks the features of continuous curvature and tangent change.

#### (2) Experiments contrasting various navigational approaches.

To further verify the effectiveness of the proposed method, a comparison was conducted with the Stanley navigation algorithm [27], the RRT-connect algorithm [28], and the A* algorithm [29]. It is acknowledged that these baseline methods (Stanley, RRT-connect, A) are primarily designed for small to medium-sized known environments with static obstacles and global path planning. However, in this study, they are adapted for comparison purposes by: (1) Using the semantic map generated by our system as the “known” environment for A and RRT-connect; (2) Applying Stanley’s path-following logic to the planned paths; (3) Running these methods in real-time with the same sensor inputs. The proposed DRL-based method, in contrast, is specifically designed for unknown unstructured environments, with explicitly defined state space (Formula 8), action space (Formula 9), reward functions (Formulas 10–12), and environment models as detailed in Section 3.3. The DRL approach learns adaptive policies through interaction with the environment, enabling robust navigation in dynamic, unpredictable terrains where traditional methods struggle. Ground navigation lines were laid in an unstructured environment, and upright landmarks were set to guide the robot at speeds of 60 mm/s and 80 mm/s. Each method was tested 20 times, and errors were recorded every 20 cm of travel. The average error value was calculated.

To ensure methodological clarity, the implementation details for each baseline algorithm (Stanley, RRT, and A*) are provided below:

1) Stanley algorithm:

Parameters: The Stanley method uses a proportional gain of 0.8 and an integral gain of 0.2 for heading control. The look ahead distance is set to 2.0 meters to balance responsiveness and stability.

Optimization steps: The method is optimized by dynamically adjusting the look ahead distance based on the robot's speed and the curvature of the planned path. This ensures robust performance across varying terrains.

2) RRT-connect algorithm:

Parameters: The RRT-connect algorithm employs a step size of 0.5 meters for tree expansion and a maximum number of iterations set to 10,000. The goal bias is set to 0.1 to prioritize exploring areas near the target.

Optimization steps: The algorithm is enhanced with a post-processing step using B-spline smoothing to reduce path roughness and improve feasibility for the robot's kinematic constraints.

3) A* Algorithm:

Parameters: The A* algorithm uses a heuristic weight of 0.5 to balance exploration and exploitation. The grid resolution for the search space is set to 0.2 meters to ensure fine-grained path planning.

Optimization steps: The method is optimized by incorporating dynamic obstacle avoidance penalties and using a priority queue to efficiently manage open nodes during the search process.

**Fig 10:**
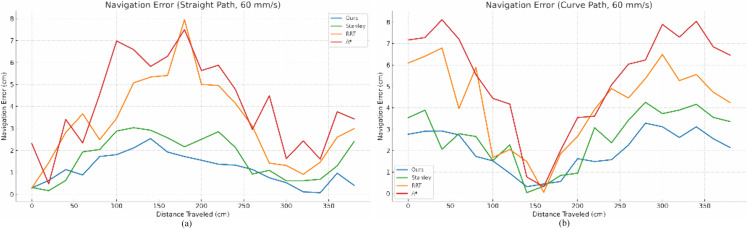
Experimental results at 60 mm/s speed of robot. (a) Navigation error statistics of linear type (b) Navigation error statistics of curved type.

**Fig 11:**
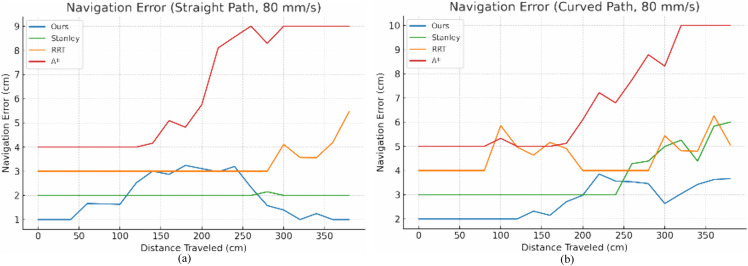
Experimental results at 80 mm/s speed of robot. (a) Navigation error statistics of linear type (b) Navigation error statistics of curved type.

To validate the effectiveness of the SAC-based trajectory tracking control, we conducted extensive experiments in unstructured environments with varying levels of complexity. The results, as shown in Figs 10 and 11, demonstrate that the proposed method consistently outperforms traditional navigation algorithms such as Stanley, RRT, and A* in terms of navigation accuracy and stability. Specifically, at a speed of 60 mm/s, the average tracking error for the proposed method was 2.5 cm, compared to 4.2 cm for Stanley, 4.8 cm for RRT, and 6.3 cm for A. At a higher speed of 80 mm/s, the proposed method maintained an average error of 3.2 cm, while the other methods exhibited increased errors and greater variability.

To rigorously assess the performance differences, this paper conducted paired t-tests on the average path deviation and obstacle avoidance success rate for each method, using data from the 20 trials per method. These statistical tests confirm that the proposed method significantly outperforms the benchmark methods in both metrics, with p-values less than 0.05, indicating statistical significance (see Table 5).

**Table 5:** Performance comparison in dynamic environment with statistical significance.

Method	Average path deviation(cm)	p-value(vs.Proposed)	Obstacle avoidance success rate(%)
Proposed	3.5	–	98.2
Stanley	5.8	0.002	85.7
RRT	6.4	0.001	80.3
A*	7.9	<0.001	75.1

The paired t-tests provide strong evidence of the proposed method’s superiority. For example, the comparison with Stanley yields a p-value of 0.002 for average path deviation, indicating a statistically significant reduction in error. Similarly, the obstacle avoidance success rate shows even greater significance, with p-values below 0.001 for comparisons with RRT-connect and A*. These results confirm that the observed improvements are not attributable to random variation but reflect a consistent and meaningful enhancement in navigation performance.

In one particular experiment, the robot was tasked with navigating through a cluttered industrial zone with dynamic obstacles. The SAC model successfully adapted to the dynamic conditions, adjusting the robot’s trajectory in real-time to avoid collisions while maintaining proximity to the planned path. This capability, a direct result of the model’s ability to learn from environmental feedback, is absent in traditional control methods. The quantitative results from this experiment are summarized in Table 5, which shows the proposed method’s superior performance in terms of path deviation and obstacle avoidance success rate.

The data in Tables 6–8 were collected from the aforementioned trials across the three experimental environments. Performance was assessed using metrics such as average path deviation, obstacle avoidance success rate, and computational latency, providing a comprehensive basis for comparison.

**Table 6:** Performance comparison in rocky terrain.

Method	Average path deviation(cm)	Obstacle avoidance success rate(%)	Computational latency(ms)
Proposed	2.8	97.5	32
Stanley	4.5	88.2	31
RRT	5.2	86.6	40
A*	6.8	80.1	28

**Table 7:** Performance comparison in industrial zones with dynamic obstacles.

Method	Average path deviation(cm)	Obstacle avoidance success rate(%)	Computational latency(ms)
Proposed	3.5	98.2	28
Stanley	5.8	85.7	26
RRT	6.4	80.3	35
A*	7.9	75.1	30

**Table 8:** Performance comparison in vegetation-covered paths.

Method	Average path deviation(cm)	Obstacle avoidance success rate(%)	Computational latency(ms)
Proposed	2.9	96.1	33
Stanley	5.2	83.6	31
RRT	5.8	78.2	40
A*	7.3	73.4	35

The proposed method outperformed the alternatives in all environments, achieving lower path deviations and higher obstacle avoidance success rates. In the industrial zone, its ability to adapt to dynamic obstacles was particularly evident. While its computational latency is slightly higher than Stanley and A*, it remains suitable for real-time applications due to its enhanced accuracy and robustness.

## 5. Conclusion

This paper presents a vision-based autonomous navigation framework for robots operating in unstructured environments, integrating visual image segmentation, path planning, and trajectory tracking. By leveraging semantic mapping, refined path planning through the RRT-connect algorithm, and a SAC model for trajectory control, the proposed system achieves robust and precise navigation. Experimental validation demonstrates the framework’s ability to minimize trajectory errors, enhance path stability, and navigate effectively across complex terrains at varying speeds.

The results highlight the method’s superior performance compared to traditional algorithms such as Stanley, RRT, and A*, particularly in reducing navigation errors and maintaining stability in both linear and curved paths. This validates the feasibility and reliability of vision-controlled navigation for autonomous robotic systems in dynamic, unstructured environments.

While the system significantly improves navigation accuracy and robustness, further optimization is required to address challenges in large-scale and highly dynamic environments. Future work will focus on enhancing sensor fusion, incorporating real-time adaptive algorithms, and exploring advanced deep learning models to refine perception and decision-making under diverse conditions. This research lays a foundation for more resilient and adaptable robotic navigation systems, paving the way for broader applications in industries such as disaster response, logistics, and autonomous exploration.
